# Males have a greater mite burden than females, and size does not matter: species- and sex-specific infestation patterns of mites (Uropodina) on burying beetles (*Nicrophorus* spp.)

**DOI:** 10.1186/s12983-026-00601-w

**Published:** 2026-02-19

**Authors:** Daria Bajerlein, Piotr Zduniak, Aleksandra Wyszyńska, Edward Baraniak, Marek Przewoźny, Tomasz Grzegorczyk, Arkadiusz Urbański

**Affiliations:** 1 Department of Animal Taxonomy and Ecology, Faculty of Biology, Adam Mickiewicz University, Uniwersytetu Poznańskiego 6, 61-614 Poznań, Poland; 2 Department of Avian Biology and Ecology, Faculty of Biology, Adam Mickiewicz University, Uniwersytetu Poznańskiego 6, 61-614 Poznań, Poland; 3 Department of Systematic Zoology, Faculty of Biology, Adam Mickiewicz University, Uniwersytetu Poznańskiego 6, 61-614 Poznań, Poland; 4 Department of Animal Physiology and Developmental Biology, Faculty of Biology, Adam Mickiewicz University, Uniwersytetu Poznańskiego 6, 61-614 Poznań, Poland

**Keywords:** Dispersal, Carrier specificity, *Nicrophorus humator*, *Nicrophorus interruptus*, *Nicrophorus vespillo*, Phoresy, Symbiosis, *Uroobovella nova*

## Abstract

**Background:**

Phoretic mites and their carriers represent a dynamic system shaped by ecological and evolutionary processes. In highly specific phoresy, which involves long-term or permanent associations, profound consequences for phoretics, including cospeciation or the transition to phoretic parasitism, can occur. Mites within the complex of cryptic species of *Uroobovella nova* are carried exclusively on burying beetles (*Nicrophorus* spp.). Nevertheless, compared with the *Poecilochirus* mite-*Nicrophorus* system, this type of interaction remains poorly understood. In this study, we investigated whether different species of burying beetles play the same role in the local dispersal of *U. nova* deutonymphs. To achieve this, we compared the infestation patterns of deutonymphs among field-collected beetle species, while accounting for sex and body size.

**Results:**

Our results revealed species-specific patterns in deutonymph infestations, with *Nicrophorus vespillo* being the most frequently infested species, followed by *N*. *humator* and *N*. *interruptus*. Furthermore, *Nicrophorus vespillo* and *N*. *humator* hosted the greatest number of deutonymphs, whereas in *N*. *interruptus,* the number of carried mites was significantly lower. The infestation pattern of *U. nova* demonstrated significant sexual bias, with males exhibiting higher mite prevalence and intensity than females. Interestingly, the variation in host body size was not a significant predictor of *U. nova* infestation. Although more mites were attached to the anterior than to the posterior parts of the beetle body in all the examined species, species- and sex-specific patterns in the distribution of deutonymphs were evident.

**Conclusions:**

Species-specific infestation patterns indicate that, at the local scale, individual burying beetle species play different roles in the dispersal of *U*. *nova* mites. Sex-specific infestation patterns suggest that biological differences between females and males may be key determinants of deutonymph infestations. Body size does not drive the prevalence, intensity, or distribution of deutonymphs. The assumption that larger hosts carry more symbionts does not hold universally in ecology.

**Supplementary Information:**

The online version contains supplementary material available at 10.1186/s12983-026-00601-w.

## Background

Phoresy is a temporary symbiosis in which a smaller animal (the phoretic or phoront) attaches to a larger animal (the carrier or host) for dispersal [1]. This behaviour is common among minute, low-mobile arthropods such as mites, which exploit ephemeral and patchily distributed resources [2]. Although the primary function of phoresy is dispersal, facilitating colonization of habitats suitable for further development, it also helps avoid overcrowding and inbreeding and reduces competition [3]. Phoresy has traditionally been regarded as a commensal interaction in which the phoront benefits from being carried without harming its host. However, increasing attention has recently been devoted to the complex nature of this relationship, which may shift along a continuum from commensalism to mutualism or parasitism, depending on the relative costs and benefits experienced by both interacting partners [4].

Phoretic interactions can range from low specificity, where a phoront uses a variety of carriers, to high specificity, where the phoront is restricted to one or a few closely related carrier species [5]. Highly specific phoresy, which involves long-term or permanent associations, can have profound consequences for phoretics, including synchronization with carrier life cycles, cospeciation, or even a transition to phoretic parasitism [1, 6, 7]. A well-studied model of highly specific phoresy is the association between mites of the genus *Poecilochirus* Canestrini and Canestrini, 1882 (Parasitidae) and burying beetles (*Nicrophorus* spp.), in which both sexes cooperate in the utilization of small vertebrate carrion as a resource for feeding and breeding [e.g., 7–13]. Deutonymphs of *Poecilochirus* attach to adult *Nicrophorus* to access their brood chambers and reproduce alongside their carriers. Depending on the ecological context, these mites may either positively or negatively influence the reproductive success of their hosts [4, 10, 11, 14].

Burying beetles also interact with mites, representing a complex of cryptic species within *Uroobovella nova* (Parasitiformes: Uropodina) (hereafter referred to as *Uroobovella nova* mites) [2, 15–17]. Unlike most species of Uropodina, which utilize a broad spectrum of carriers, including representatives of several coleopteran families, *U*. *nova* deutonymphs are transported exclusively by burying beetles [18]. Like *Poecilochirus carabi*, *U*. *nova* was initially considered a generalist with a broad range of burying beetle hosts. However, Knee et al. [16] demonstrated that *U*. *nova* comprises at least five cryptic species associated with different *Nicrophorus* species. The true nature of the phoretic associations between *U*. *nova* mites and their hosts remains poorly understood.

A fundamental step in understanding any phoretic interaction is to determine the carrier range of the phoronts. This involves identifying the carriers and assessing the level of mite infestation on each carrier to distinguish primary carriers from incidental carriers. Beetles of the genus *Nicrophorus* are distributed worldwide and comprise approximately 65 species [19–21]. Eleven species of *Nicrophorus* have been reported from Europe [22], including eight from Poland [23, 24]. In a previous study [17], we presented findings on the symbiosis between *U. nova* mites and the common European burying beetle *Nicrophorus vespilloides* Herbst, 1783. Most notably, we reported a high mite prevalence of approximately 90%, with a mean infestation intensity of 24.5 deutonymphs per beetle. We also observed strong mite specificity in the selection of attachment sites, a positive but weak relationship between the number of carried deutonymphs and beetle body size, and a slight deutonymph preference for female beetles [17].

In this study, we further examined the *Uroobovella nova*–*Nicrophorus* system, focusing on three additional, less abundant burying beetle species recorded during our research: *Nicrophorus vespillo* (Linnaeus, 1758), *Nicrophorus humator* (Gleditsch, 1767), and *Nicrophorus interruptus* Stephens, 1830. Among European *Nicrophorus* beetles, *N. vespilloides* and *N*. *vespillo* are the most frequently reported carriers of *U*. *nova* mites [2, 15–17, 25, 26]. Deutonymphs have been recorded less commonly in other burying beetle species, such as *N*. *humator* [2, 15]. The primary objective of this study was to assess whether *N*. *vespillo*, *N*. *humator*, and *N*. *interruptus* play as important a role as carriers of *U*. *nova* mites as *N. vespilloides* does. To address this, we quantified phoretic deutonymph infestations by analysing both the prevalence and intensity of infestations across beetle species, as well as the potential effects of beetle sex and body size. Additionally, we examined whether the distribution patterns of mites on the studied *Nicrophorus* species corresponded to those previously reported for *N. vespilloides*.

## Materials and methods

### Fieldwork

Burying beetles were obtained as bycatch in 2018 and 2019 from traps originally deployed for saproxylic beetles in the Niepołomice Forest—a large woodland area located near Kraków, southern Poland (49°59′–50°07′ N, 20°13′–20°28′ E; covering approximately 110 km^2^). The trapping method used was the IBL-2 flight interception trap, which consisted of a triangular screen and a funnel attached to a container filled with ethylene glycol, which served as a preservative. Immersion of beetles in ethylene glycol prevented the movement of attached mites between hosts, thereby allowing examination of infestation patterns.

Beetles with attached mites were collected during four sampling periods: 30 May–12 July 2018, 13 July–18 September 2018, 18 May–2 July 2019, and 3 July–5 September 2019. For the analysis, we used 880 beetles, including 275 specimens of *N*. *humator*, 315 of *N*. *interruptus*, and 290 of *N*. *vespillo*, all collected between 13 July and 18 September 2018. The number of beetles collected during the other sampling periods was too low and was thus excluded from the analysis. The numbers of beetles collected during each sampling period are provided in Supplementary Table 1.

### Ecology of the studied beetles

*Nicrophorus humator*, *N*. *interruptus*, and *N*. *vespillo* are Palearctic species distributed from Europe and North Africa eastwards to Siberia and northwestern China [27]. Among Polish burying beetles, *N*. *vespillo* and *N*. *humator* are, along with *N. vespilloides*, the most widespread species, whereas *N*. *interruptus* occurs more frequently in southern Poland [24, 28].

*Nicrophorus vespillo* is active from March to October, with activity peaks occurring either in early spring or mid-summer, depending on geographic location [22, 29–33]. This species typically reproduces up to three times per year, with the last breeding period occurring in August [31]. *Nicrophorus vespillo* is active from noon to midnight, with peak activity in the afternoon [31]. It prefers open habitats such as meadows, fields, wetlands, and sandy grasslands [29, 30, 34–36] but is also occasionally found at forest edges [32]. Individuals most frequently appear on carcasses around the fifth day of exposure and are attracted equally to both smaller (mice) and larger (rats) carrion [32].

*Nicrophorus humator* is active from April to October, with two activity peaks in April–May and August–October [29, 30, 32, 33]. It is considered a typical forest species [22, 29, 30, 32, 36], although it is also found in nonforest habitats, including field margins, forest edges, and sandy grasslands [29, 30, 32]. The species breeds up to three times per year, with beetles observed in October representing young individuals from the last generation [31]. Its activity extends from the afternoon until midnight, peaking after dusk [31]. *Nicrophorus humator* is most frequently recorded on small carcasses, such as those of mice [32, 37].

In Europe, *N*. *interruptus* occurs from March to October, with activity peaking in summer [22, 33, 38]. It is regarded as a primarily forest-dwelling species, but it also inhabits open and semiopen areas, including extensively used wetlands and fields [22, 39, 40]. According to Jakubec and Růžička [27], *N*. *interruptu*s is a eurytopic species that occurs in both forested and open landscapes. Its diurnal activity is predominantly crepuscular [22]. The preferences of *Nicrophorus interruptus* for carcass size remain poorly understood.

### Analysis of mite-beetle interactions

All collected beetle specimens were examined for phoretic deutonymphs of *U*. *nova* mites and their pedicels. The pedicel is a temporary, stalk-like structure with two extended termini [41–43]. One terminus attaches to the deutonymph’s anal region, whereas the other attaches to the carrier’s body surface. After deutonymph detachment, the pedicel typically remains on the carrier as evidence of the mite’s prior presence.

For each beetle, the number of deutonymphs and pedicels without deutonymphs, as well as their locations on the host’s body, were recorded. Phoretic deutonymphs were identified via the Uropodina identification key by Karg [44]. Burying beetles were identified according to keys for the identification of burying beetles by Mroczkowski [45], Jałoszyński [46] and UK Beetle recording website [47] and sexed by genital dissection [48]. Beetle body size was determined by measuring pronotum width, defined as the distance between the two most lateral points, following Jarrett et al. [49].

### Data analysis

The differences in body size between beetles were calculated with the Factorial ANOVA, where SPECIES, SEX, and SPECIES*SEX were the factors. Furthermore, we analysed the influence of beetles’ SPECIES, SEX, and SIZE as well as the interaction effect of SPECIES*SEX on deutonymph prevalence (the proportion of infested beetles) via a Generalized Linear Model (GLM) with a binomial distribution and logit link function, where prevalence (binary variable) was the dependent variable and SPECIES (categorical variable), SEX (binary variable), and SIZE (continuous variable) were the factors. Moreover, we analysed the impacts of beetles’ SPECIES, SEX and SIZE, as well as the interaction effects of SPECIES*SEX on the intensity of deutonymph infestation, e.g., the mean number of deutonymphs per infested beetle, via a GLM with a Poisson distribution and a log link function, where the intensity of mite infestation was the dependent variable and SPECIES, SEX, SIZE, and SPECIES*SEX were the factors. Finally, we examined the distribution of mites on beetles, considering the most infested parts of their bodies [PART], taking into account their left or right side (a total of eleven body parts), SPECIES, SEX, and body SIZE via a Generalized Linear Mixed Model (GLMM) with a Poisson distribution and log link function, where the number of mites was the dependent variable; SPECIES, SEX, PART, body SIZE, and SPECIES*SEX*PART were fixed factors; and the beetle ID was a random factor. All calculations were performed via IBM SPSS Statistics for Windows [50]. Throughout the text, the mean values are presented with 95% confidence limits (CLs).

**Fig. 1:**
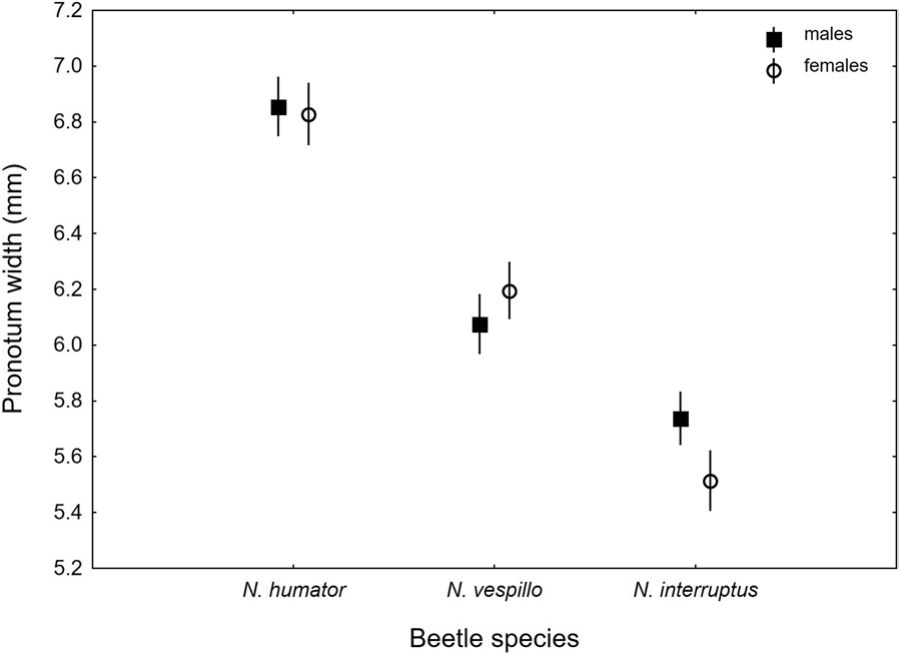
Body size expressed as pronotum width in *Nicrophorus humator*, *N*. *vespillo*, and *N*. *interruptus,* including their sex; mean values are presented with 95% confidence limits

Significant differences in body size were determined based on pronotum width among the collected SPECIES. *Nicrophorus humator* was the largest, *N*. *interruptus* smallest, and *N*. *vespillo* of intermediate size (Fig. 1, Supplementary Table 2). The effect of sex was insignificant, but we found a significant interaction of SPECIES and SEX, with larger males in *N*. *interruptus*, and no intersexual differences in the other species (Fig. 1, Supplementary Table 2).

## Results

### Deutonymph prevalence

**Fig. 2:**
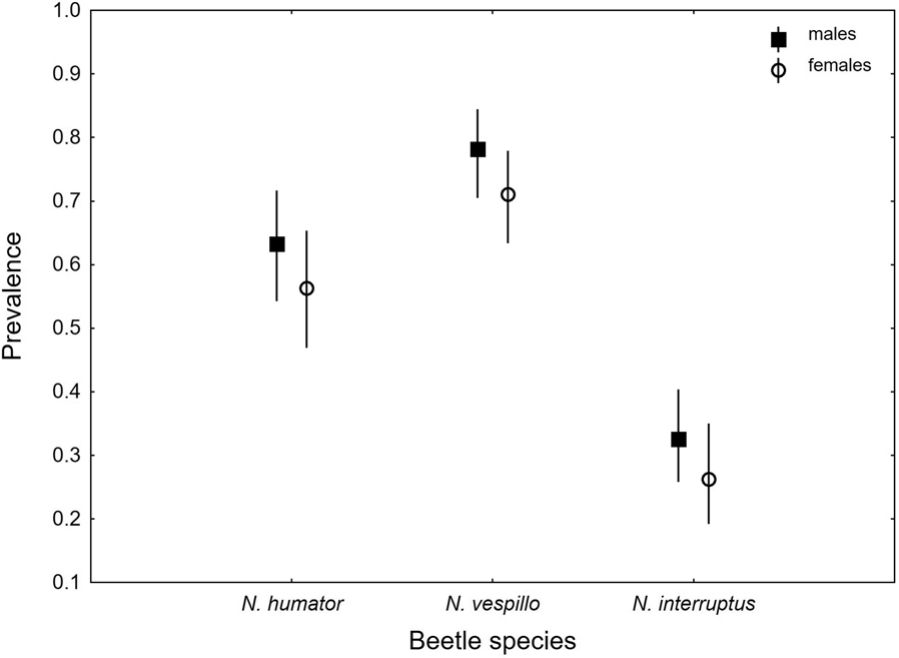
Prevalence of deutonymphs within the *Uroobovella nova* complex of cryptic species carried by *Nicrophorus humator*, *N*. *vespillo*, and *N*. *interruptus* in relation to sex; mean values are presented with 95% confidence limits

The infestation status of the beetles varied between SPECIES, with *N*. *vespillo* being the most frequently infested and *N*. *interruptus* the least frequently infested (Tables 1, 2, Fig. 2). The effect of SEX was also significant, with a relatively high deutonymph prevalence reported in males (Tables 1, 2, Fig. 2). The deutonymph prevalence did not depend on beetles’ SIZE, and the interaction effect of SPECIES and SEX was also insignificant (Tables 1, 2, Fig. 2).

**Table 1:** Summary of Generalized Linear Model (GLM) analysis of factors influencing the prevalence of deutonymphs from the *Uroobovella nova* complex of cryptic species on *Nicrophorus humator*,* N*. *vespillo*, and* N*.* interruptus*

Factor	Wald χ^2^	df	*p*
INTERCEPT	0.595	1	0.441
SPECIES	101.888	2	< 0.001
SEX	4.765	1	0.029
SPECIES*SEX	0.058	2	0.971
BODY SIZE	0.242	1	0.623

**Table 2:** Prevalence and intensity of infestation of deutonymphs within the *Uroobovella nova* complex of cryptic species of mites phoretic on burying beetles

Beetle species	N	K	P (%)	95% CL	I ± SD	Min–Max
***Nicrophorus humator*(Gleditsch)**	275	163	59.27	53.21–65.13	19.43 ± 27.53	1–147
*Females*	133	75	56.39	47.53–64.97	15.96 ± 26.31	1–138
*Males*	142	88	61.97	53.45–69.98	22.39 ± 28.34	1–147
***Nicrophorus vespillo*** **(Linnaeus)**	290	216	74.48	69.06–79.40	18.58 ± 27.74	1–192
*Females*	152	108	71.05	63.15–78.11	15.28 ± 18.95	1–105
*Males*	138	108	78.26	70.44–84.83	21.88 ± 34.14	1–192
*** Nicrophorus interruptus*** **Stephens**	315	96	30.48	25.44–35.88	4.88 ± 5.26	1–28
*Females*	138	37	26.81	19.63–35.01	4.78 ± 5.19	1–28
*Males*	177	59	33.33	26.44–40.80	4.93 ± 5.35	1–26

N, The number of collected beetles; K, The number of infested beetles; P, Prevalence; I, The intensity of mite infestation; CL, Confidence limits; SD, Standard deviation; Min–Max, The minimal and maximal number of carried mites

### Intensity of deutonymph infestation

**Fig. 3:**
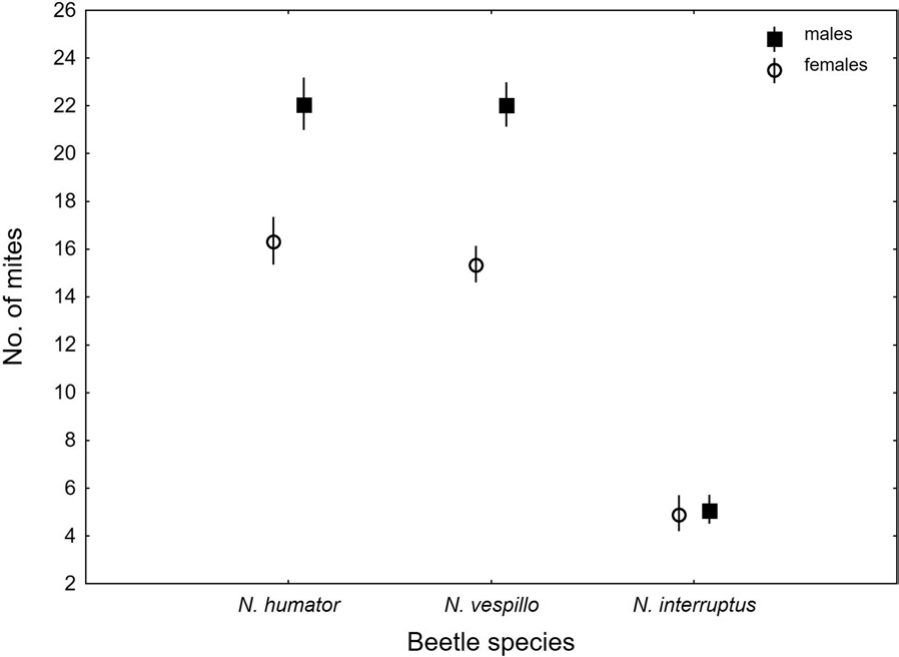
Intensity of infestation of deutonymphs within the *Uroobovella nova* complex of cryptic species carried by *Nicrophorus humator*, *N*. *vespillo*, and *N*. *interruptus* in relation to sex; mean values are presented with 95% confidence limits

The intensity of deutonymph infestation differed significantly among SPECIES, being the highest on *N*. *vespillo* and *N*. *humator* and the lowest on *N*. *interruptus* (Tables 2, 3, Fig. 3). The number of mites was positively related to beetles’ SEX, with males carrying a higher mite load (Tables 2, 3, Fig. 3). The interaction effect between SPECIES and SEX was also significant; males of *N*. *vespillo* and *N*. *humator* carried more mites, whereas no sex-related differences were detected in *N*. *interruptus* (Tables 2, 3, Fig. 3). The SIZE of the beetle did not affect the intensity of the deutonymph infestation (Tables 2, 3).

**Table 3:** Summary of the Generalized Linear Model (GLM) analysis of factors influencing the intensity of infestation of deutonymphs from the *Uroobovella nova* complex of cryptic species on *Nicrophorus humator, N. vespillo,* and* N. interruptus*

Factor	Wald χ^2^	df	*p*
INTERCEPT	424.440	1	< 0.001
SPECIES	677.253	2	< 0.001
SEX	42.879	1	< 0.001
SPECIES*SEX	10.713	2	0.005
BODY SIZE	2.356	1	0.125

### Localization of deutonymphs on the beetles’ bodies

**Fig. 4:**
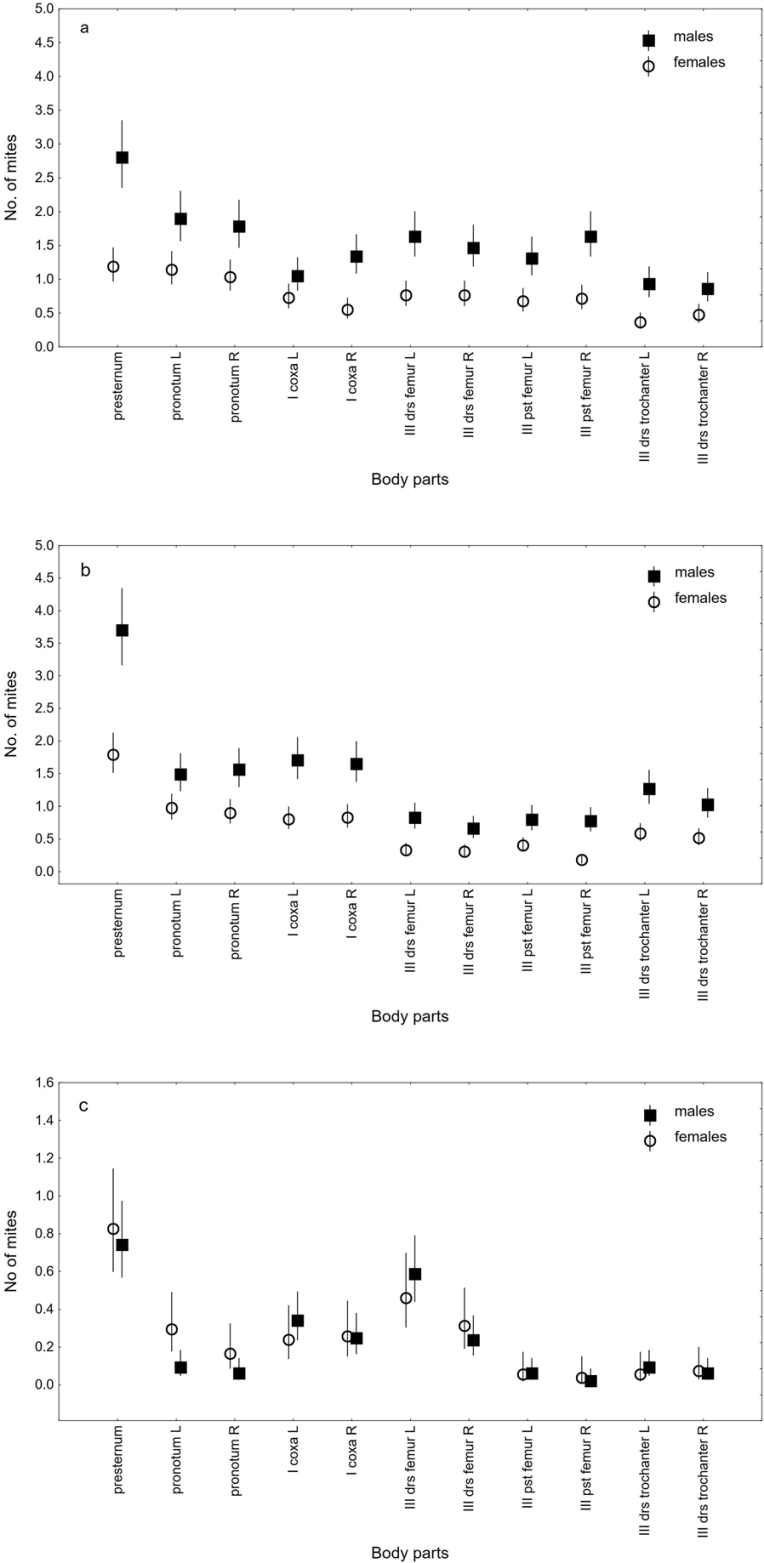
Mean numbers of deutonymphs per infested beetle within the *Uroobovella nova* complex of cryptic species attached to body parts of *Nicrophorus humator* (**a**), *N*. *vespillo* (**b**), and *N*. *interruptus* (**c**). Legend: L, R–the left and right body sides, presternum–the prothorax presternum, I–forelegs, III–hindlegs, pronotum–ventral parts of pronotum lateral margins, coxa–the ventral side of the coxae, drs–the dorsal side, pst–the posterior side; the means are presented with 95% confidence limits

The analysis of the most heavily infested beetles’ body PARTS (n = 11), considering both the left and right sides of the body as well as SPECIES, SEX, body PARTS, body SIZE, and the interaction effect of SPECIES*SEX*PARTS, revealed differences in deutonymph location on their carriers (Table 4, Fig. 4).

**Table 4:** Summary of the Generalized Linear Mixed Model (GLMM) analysis of factors influencing the attachment patterns of deutonymphs from the *Uroobovella nova* complex of cryptic species on *Nicrophorus humator, N. vespillo,* and* N. interruptus*

Factor	F	df1	df2	*p*
SPECIES	215.793	2	5114	< 0.001
SEX	47.719	1	5114	< 0.001
BODY PARTS	64.425	10	5114	< 0.001
BODY SIZE	2.034	1	5114	0.154
SPECIES*SEX*BODY PARTS	9.922	52	5114	< 0.001
MODEL	36.170	66	5114	< 0.001

**Fig. 5:**
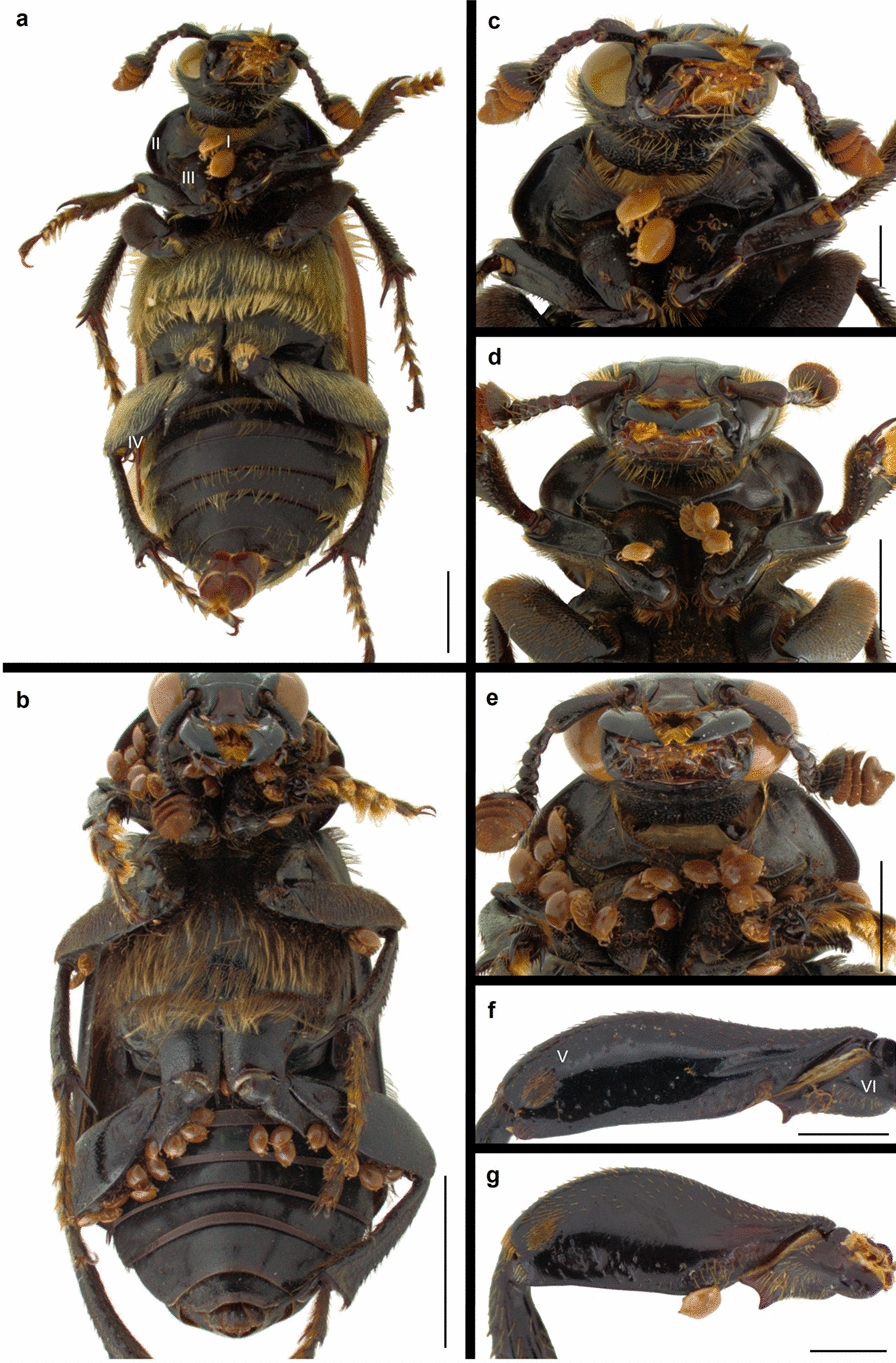
Burying beetles with attached deutonymphs and pedicels of the *Uroobovella nova* complex of cryptic species. Legend: **a** Localization of mites on the ventral side of *Nicrophorus vespillo*; **b** Localization of mites on the ventral side of *N*. *humator*; **c** Mites attached to the prothorax presternum and foreleg coxae of *N*. *vespillo*; **d** Mites attached to the prothorax presternum and foreleg coxae of *N*. *interruptus*; **e** Mites attached to the prothorax presternum, pronotum lateral margins, and foreleg coxae of *N*. *humator*; **f**, **g** Pedicels and deutonymphs attached to the dorsal femora and trochanters of hindlegs in *N*. *vespillo* (**f**) and *N*. *interruptus* (**g**). I–prothorax presternum; II–pronotum lateral margin, III–foreleg coxa, IV–posterior part of the femur of the hindleg, V–dorsal part of the femur of the hindleg, VI–dorsal part of the trochanter of the hindleg. Scale bars: 2 mm (**a**, **d**, **e**); 5 mm (**b**); 1 mm (**c**, **f**, **g**)

The effect of SPECIES was significant, with greater numbers of deutonymphs recorded on particular body parts in *N. humator* and *N. vespillo* than in *N. interruptus* (Table 4, Fig. 4). In *N. humator*, the distribution of mites was relatively uniform, with smaller differences between the numbers of deutonymphs attached to the presternum, the lateral sides of the ventral pronotum, and the coxae of the forelegs. In contrast, in *N. vespillo,* the difference between the intensity of mite infestations on the presternum and other body parts was more pronounced. In *N. interruptus*, deutonymphs, in addition to their distinct preference for the presternum, also attach particularly frequently to the left femora of hindlegs (Table 4, Figs. 4, 5). SEX significantly affected mite distribution, with males exhibiting more heavily infested body parts than females. The most pronounced intersexual differences in the intensity of deutonymph infestation were observed within the presternum (Table 4, Figs. 4, 5). The effect of body PARTS was significant, with more deutonymphs attached to anterior regions such as the presternum, lateral sides of the ventral pronotum, and coxae of the forelegs than to posterior body parts, particularly the femora and trochanters of the hindlegs. Deutonymphs were evenly distributed between the left and right sides of the host body, except for the femora of the hindlegs in *N*. *interruptus* (Table 4, Fig. 4). The interaction effect of SPECIES, SEX, and PARTS was also significant, with males of *N*. *humator* and *N*. *vespillo* carrying more deutonymphs on the anterior body regions of the hosts. In *N*. *interruptus*, differences in mite distribution were less consistent and similar between the sexes. Moreover, in females of *N*. *humator* and *N*. v*espillo*, deutonymphs were distributed more evenly across the examined body regions. In contrast, in males, a more distinct preference for the presternum was observed (Table 4, Figs. 4, 5). The effect of body SIZE on mite localization was not significant (Table 4).

## Discussion

In this work, we present novel and significant findings on the highly specific, phoretic interactions between *U*. *nova* mites and burying beetles (*Nicrophorus* spp.). *Nicrophorus vespilloides*—previously investigated by us [17]—exhibits the highest level of infestation, followed by *N*. *vespillo* and *N*. *humator*. In contrast, *N*. *interruptus* has the lowest infestation rate and has not yet been reported as a carrier of *U*. *nova* mites.

There may be several reasons for the variation in *U*. *nova* mite infestations among the studied beetles. First, the question arises whether all burying beetles studied carry the same mite species within the analysed mite complex. Knee et al. [16] reported that in Europe, *N. vespilloides* and *N*. *vespillo* carry *Uroobovella* sp. 3. In turn, Athias-Binche et al. [2] reported that deutonymphs collected from *N*. *vespillo* and *N. vespilloides* are smaller than those collected from *N*. *humator*, which may indicate that they are separate morphotypes. Research conducted by Kočárek [31] demonstrated that Horn’s index, which measures niche overlap, remains consistently high for *N. vespilloides* and *N*. *vespillo* throughout their entire seasonal activity. Owing to differences in diurnal activity and habitat preference, Horn’s index is very low in spring between *N*. *vespillo* and *N*. *humator* as well as between *N. vespilloides* and *N*. *humator* but increases significantly during summer and autumn. A high degree of niche overlap between *N. vespilloides* and *N*. *vespillo* may facilitate possible host switching in *U*. *nova* mites, in contrast to the lower niche overlap observed between these two species and *N*. *humator*. Different *Nicrophorus* species may compete for the same carcass [51, 52], creating opportunities for interspecific switching between hosts in phoretic deutonymphs. These events could promote gene flow among mite populations and hinder speciation. However, host switching on carcasses seems unlikely, as, according to previous observations [41], deutonymphs of Uropodina detach only in suitable microhabitats, where their development occurs. Attachment via a pedicel further limits movement between hosts. Thus, host switching probably occurs mainly when a breeding chamber is used by another *Nicrophorus* species, allowing deutonymphs of the next generation to disperse with the new host’s offspring. Most likely, the deutonymphs examined in our study represent a single species, *Uroobovella* sp. 3, distinguished by Knee et al. [16], with the possible occurrence of host races associated with *N*. *humator* and perhaps *N*. *interruptus*. Therefore, it is likely that the observed variation in deutonymph infestation among the examined beetles results not from their different representative species within the studied complex but rather from interspecific differences in the biology of their carriers, particularly in their breeding behaviour. Unfortunately, these behaviours have been poorly studied in *N*. *humator* and *N*. *interruptus*, making it difficult to draw any firm conclusions at this stage. The susceptibility of the carrier to infestation by Uropodina mites may also be shaped by other host-dependent factors, such as seasonal activity and population dynamics, duration of exposure to mites, morphology of the host body surface, and host behaviors related to grooming [for a review, see 18]. Infestation is also influenced by mite-dependent factors, such as their seasonal activity, population dynamics, body size, and method of attachment [for a review, see 18].

In the studied *Uroobovella*–*Nicrophorus* system, carrier body size does not appear to be a factor driving mite prevalence or load. Although our previous study demonstrated that the body size of *N. vespilloides* positively affects the intensity of deutonymph infestation, the effect was weak and only marginally significant. *Nicrophorus vespilloides*, despite being the smallest among the studied burying beetle species (mean pronotum width of 4.92 mm [17]), was more heavily infested than the much larger *N*. *humator* examined in this study. In this context, our results align with previous findings suggesting that body size does not play a consistently significant role in determining host suitability for symbionts [e.g., 53–55].

The effect of beetle sex on prevalence was significant, with males being more frequently selected as carriers than females. In *N*. *vespillo* and *N*. *humator*, males carried a higher mite load, whereas in *N*. *interruptus,* no effect of sex on the number of carried deutonymphs was observed. For *N. vespilloides*, we recorded a slightly greater mite load in females (8.1% more mites than in males) [17]. These sex-specific infestation patterns are likely related to differences in breeding biology between sexes across the studied species. Cooperation between males and females in the preparation of brood chambers and in parental care has been well documented in *N. vespilloides* [56, 57] and *N. vespillo* [58] but not in the other *Nicrophorus* species examined. Therefore, further research is needed to explore the breeding behaviour of burying beetles in more detail.

Although the general pattern of *U*. *nova* mite distribution was similar among the *Nicrophorus* species examined, with more individuals occurring in the anterior body regions, particularly the prothorax presternum, regardless of host body size (consistent with our previous findings for *N. vespilloides*), our results revealed species- and sex-specific differences in the intensity of deutonymph infestation across the studied body parts. In *N*. *humator*, deutonymphs were distributed more evenly across all body parts. In contrast, in *N*. *vespillo,* differences observed between the number of mites attached to the presternum and those on other body parts were more distinct. In *N*. *interruptus*, the overall number of deutonymphs was very low and evenly distributed across the body, except for the dorsal side of the femora of the left hindlegs. In *N*. *vespillo* and *N*. *humator*, pronounced intersexual differences in deutonymph distribution were recorded, but these differences were not detected in *N*. *interruptus*. In the previously studied *N. vespilloides*, no effect of sex on the distribution of mites was found, and the coxae of the first pair of legs were, after the presternum, the second most heavily colonized body part [17]. The observed variation in deutonymph distribution patterns most likely results from interspecific and intersexual differences in host biology, which remain poorly understood for the species studied here. The spatial distribution of Uropodina mites on the host likely affects their dispersal success, with mites possibly attaching to areas that lower the risk of detachment and do not hinder host movement [e.g., 59–61].

Our study provides new and intriguing insights into the highly specific symbiosis between *U. nova* and burying beetles, complementing previous research on this interaction. Future studies should address local genetic differentiation within the *U. nova* species complex to clarify its taxonomic status. In addition, laboratory experiments are needed to observe the biology of *U. nova* mites within the breeding chambers of burying beetles, their impact on their hosts, and their interactions with *Poecilochirus* mites, thereby contributing to a better understanding of how phoretic symbiosis is shaped in mites coevolving with their hosts in carrion-associated systems.

## Conclusions

We showed that at the local scale, *U*. *nova* mites use different burying beetle species for dispersal and may exhibit host-specific infestation patterns. These findings suggest that individual burying beetle species may play different roles in the dispersal of deutonymphs. The previously studied *N. vespilloides* is the primary carrier of *U*. *nova* deutonymphs, followed by *N*. *vespillo* and *N*. *humator* analysed in the present study, whereas *N*. *interruptus* plays a considerably less significant role. We also observed sex-specific infestation patterns, which appeared more consistent in terms of prevalence but more variable in terms of the intensity of mite infestation. Interestingly, variation in host body size was not a significant predictor of *Uroobovella nova* infestation. This finding demonstrates that the simple correlation “larger hosts carry more symbionts” does not hold universally in ecology. Although more mites are attached to the anterior than to the posterior parts of the beetle body in all the examined species, species- and sex-specific patterns in the distribution of deutonymphs were evident. Multiple factors likely influence differences in infestations among burying beetle species; in our view, beetle species- and sex-specific breeding behaviours may play a key role in shaping the infestation patterns of *U*. *nova* mites.

## Supplementary Information

Additional file1 (DOCX 24 KB)

## Data Availability

The datasets used during the current study are available from the corresponding author upon reasonable request.

## References

[1] Seeman OD, Walter DE. Phoresy and mites: more than just a free ride. Annu Rev Entomol. 2023;68:69–88. 36170643 10.1146/annurev-ento-120220-013329

[2] Athias-Binche F, Schwarz HH, Meierhofer I. Phoretic association of *Neoseius novus* (Ouds., 1902) (Acari: Uropodina) with *Nicrophorus* spp. (Coleoptera: Silphidae): a case of sympatric speciation? Int J Acarol. 1993;19:75–86.

[3] Athias-Binche F. Dispersal in varying environments: the case of phoretic uropodid mites. Can J Zool. 1993;71:1793–8.

[4] Sun S-J. A framework for using phoresy to assess ecological transition into parasitism and mutualism. Symbiosis. 2022;86:133–8.

[5] Athias-Binche F. Evolutionary ecology of dispersal in mites. In: Dusbábek F, Bukva V, editors. Modern Acarology, vol. 1. The Hague: Academia, Prague and SPB Academic Publishing bv; 1991. p. 27–41.

[6] Athias-Binche F, Morand S. From phoresy to parasitism: the example of mites and nematodes. Res Rev Parasitol. 1993;53:73–9.

[7] Canitz J, Sikes DS, Knee W, Baumann J, Haftaro P, Steinmetz N, et al. Cryptic diversity within the *Poecilochirus carabi* mite species complex phoretic on *Nicrophorus* burying beetles: phylogeny, biogeography, and host specificity. Mol Ecol. 2022;31:658–74. 34704311 10.1111/mec.16248

[8] Grossman JD, Smith RJ. Phoretic mite discrimination among male burying beetle (*Nicrophorus investigator*) hosts. Ann Entomol Soc Am. 2008;101:266–71.

[9] De Gasperin O, Duarte A, Kilner RM. Interspecific interactions explain variation in the duration of paternal care in the burying beetle. Anim Behav. 2015;109:199–207. 26778845 10.1016/j.anbehav.2015.08.014 PMC4686539

[10] Nehring V, Teubner H, König S. Dose-independent virulence in phoretic mites that parasitize burying beetles. Int J Parasitol. 2019;49:759–67. 31401062 10.1016/j.ijpara.2019.05.011

[11] Sun S-J, Kilner RM. Temperature stress induces mites to help their carrion beetle hosts by eliminating rival blowflies. Elife. 2020;9:e55649. 32755542 10.7554/eLife.55649 PMC7431131

[12] Sun S-J, Kilner RM. Competition among host-specific lineages of *Poecilochirus carabi* mites influences the extent of co-adaptation with their *Nicrophorus vespilloides* burying beetle hosts. Ecol Evol. 2024;14:e10837. 38192905 10.1002/ece3.10837 PMC10771929

[13] Lan B, Malik TG, Tsai M-T, Wu Y-T, Sun S-Y. No evidence that the phoretic mite *Poecilochirus carabi* influences mate choice or fitness in the host burying beetle *Nicrophorus nepalensis*. Ecol Evol. 2025;15:e71733. 40625334 10.1002/ece3.71733 PMC12231222

[14] De Gasperin O, Kilner RM. Interspecific interactions and the scope for parent-offspring conflict: high mite density temporarily changes the trade-off between offspring size and number in the burying beetle, *Nicrophorus vespilloides*. PLoS ONE. 2016;11:e0150969. 10.1371/journal.pone.0150969 PMC4795767 26985819

[15] Schwarz HH, Starrach M, Koulianos S. Host specificity and permanence of associations between mesostigmatic mites (Acari: Anactinotrichida) and burying beetles (Coleoptera: Silphidae: *Nicrophorus*). J Nat Hist. 1998;32:159–72.

[16] Knee W, Beaulieu F, Skevington JH, Kelso S, Cognato AI, Forbes MR. Species boundaries and host range of tortoise mites (Uropodoidea) phoretic on bark beetles (Scolytinae), using morphometric and molecular markers. PLoS ONE. 2012;7:e47243. 23071768 10.1371/journal.pone.0047243 PMC3469529

[17] Bajerlein D, Zduniak P, Wyszyńska A, Baraniak E, Przewoźny M, Grzegorczyk T, et al. Influences of carrier sex, body size, and time on the symbiotic interaction between *Nicrophorus vespilloides* and the *Uroobovella nova* mite species complex. Sci Rep. 2025;15:19823. 40473732 10.1038/s41598-025-04685-y PMC12141562

[18] Bajerlein D, Błoszyk J, Halliday B, Konwerski S. Hitchhiking through life: a review of phoresy in Uropodina mites (Parasitiformes: Mesostigmata). Eur Zool J. 2024;91:31–63.

[19] Sikes DS, Madge RB, Newton AF. A catalog of the Nicrophorinae (Coleoptera: Silphidae) of the world. Zootaxa. 2002;65:1–304.

[20] Sikes DS, Vamosi SM, Trumbo TS, Ricketts M, Venables C. Molecular systematics and biogeography of *Nicrophorus* in part—the *investigator* species group (Coleoptera: Silphidae) using mixture model MCMC. Mol Phylogenet Evol. 2008;48:646–66. 18562216 10.1016/j.ympev.2008.04.034

[21] Sikes D, Trumbo TS, Peck SB. Cryptic diversity in the New World burying beetle fauna: *Nicrophorus hebes* Kirby; new status as a resurrected name (Coleoptera: Silphidae: Nicrophorinae). Arthropod Syst Phylogeny. 2016;74:299–309.

[22] Dekeirsschieter J, Verheggen F, Lognay G, Haubruge E. Large carrion beetles (Coleoptera, Silphidae) in Western Europe: a review. Biotechnol Agron Soc Environ. 2011;15:435–47.

[23] Aleksandrowicz O, Komosiński K. On the fauna of carrion beetles (Coleoptera, Silphidae) of Mazurian lakeland (north-eastern Poland). In: Skłodowski J, Huruk S, Barševskis A, Tarasiuk S, editors. Protection of Coleoptera in the Baltic sea region. Warsaw Agricultural University Press; 2005. p. 147–53.

[24] Konieczna K, Czerniakowski Z, Wolański P. The occurrence and species richnes of nicrophagous Silphidae (Coleoptera) in wooded areas in different degree of urbanization. Baltic J Coleopterol. 2019;19:213–32.

[25] Wiśniewski J. Für die Fauna Polens neue Uropodina (Acari: Parasitiformes). Teil. II. Fragm Faun. 1982;27:143–7.

[26] Mašán P. Mites of the cohort Uropodina (Acarina, Mesostigmata) in Slovakia. Annot Zool Bot. 2001;223:1–320.

[27] Jakubec P, Růžička J. Distribution of open landscape carrion beetles (Coleoptera: Silphidae) in selected lowlands of the Czech Republic. Klapalekiana. 2012;48:169–89.

[28] https://www.gbif.org

[29] Růžička J. Seasonal activity and habitat associations of Silphidae and Leiodidae: Cholevinae (Coleoptera) in central Bohemia. Acta Soc Zool Bohem. 1994;58:67–78.

[30] Kočárek P, Benko K. Výskyt a sezónní aktivita brouků čeledi Silphidae na Hlučínsku (Slezsko, Česká republika). Occurrence and distribution of Silphidae in Hlučín region (Silesia, Czech Republic). Čas Slez Muz Opava. 1997;46:173–9.

[31] Kočárek P. Diurnal activity rhythms and niche differentiation in a carrion beetle assemblage (Coleoptera: Silphidae) in Opava, the Czech Republic. Biol Rhythm Res. 2001;32:431–8.

[32] Urbański A, Baraniak E. Differences in early seasonal activity of three burying beetle species (Coleoptera: Silphidae: *Nicrophorus* F.) in Poland. Coleopt Bull. 2015;69:283–92.

[33] Vrezec AL, Ergaver ŠA, Kapla A, Ratajc U. Material for the beetle fauna (Coleoptera) of Slovenia, 5th contribution: Polyphaga: Staphyliniformia: Staphylinoidea: Silphidae. Scopolia. 2020;99:1–153.

[34] Pukowski E. Ökologische untersuchungen an *Necrophorus* F. Zoomorphology. 1933;27:518–86.

[35] Otronen M. The effect of body size on the outcome of fights in burying beetles (*Nicrophorus*). Ann Zool Fenn. 1988;25:191–201.

[36] Scott MP. The ecology and behavior of burying beetles. Annu Rev Entomol. 1998;43:595–618. 15012399 10.1146/annurev.ento.43.1.595

[37] Anderson RS. Resource partitioning in the carrion beetle (Coleoptera: Silphidae) fauna of southern Ontario: ecological and evolutionary considerations. Can J Zool. 1982;60:1314–25.

[38] Catherall-Ostler AM. Size-based niche partitioning permits coexistence in natural populations of *Nicrophorus* spp. Environ Entomol. 2025;54:1412–22. 41147415 10.1093/ee/nvaf087 PMC12716276

[39] Konieczna K, Czerniakowski W, Olbrycht T. Materiały do poznania zgrupowań chrząszczy omarlicowatych (Col., Silphidae) w uprawach ziemniaka i biocenozach leśnych wybranych regionów Polski południowo-wschodniej. Episteme. 2014;22:173–84.

[40] Pietraszko M, Warchałowski M. Materiały do poznania zgrupowań chrząszczy omarlicowatych (Coleoptera: Silphidae) na terenie Beskidu i Pogórza Śląskiego. Acta Entomol Slov. 2017;25:1–7.

[41] Faasch H. Beitrag zur Biologie der einheimischen Uropodiden *Uroobovella marginata* (CL Koch 1839) und *Uropoda orbicularis* (OF Müller 1776) und experimentelle Analyse ihres Phoresieverhaltens. Zool Jahrb Abt Syst. 1967;94:521–608.

[42] Bajerlein D, Witaliński W. Anatomy and fine structure of pedicellar glands in phoretic deutonymphs of uropodid mites (Acari: Mesostigmata). Arthropod Struct Dev. 2012;41:245–57. 22406081 10.1016/j.asd.2012.02.006

[43] Bajerlein D, Witaliński W, Adamski Z. Morphological diversity of pedicels in phoretic deutonymphs of Uropodina mites (Acari: Mesostigmata). Arthropod Struct Dev. 2013;42:185–96. 23454448 10.1016/j.asd.2013.02.002

[44] Karg W. Acari (Acarina), Milben. Unterordnung Parasitiformes (Anactinochaeta). Uropodina Kramer, Schlidkrötenmilben. Die Tierwelt Deutschlands. VEB Gustav Fischer Verlag, Jena. 1989;67:1–203.

[45] Mroczkowski M. Omarlicowate—Silphidae. Klucze do oznaczania owadów Polski. Warszawa, XIX, 1955; 25:1–29.

[46] Jałoszyński P. https://entomo.pl/artykuly/abcjaloszynski/index.php

[47] https://coleoptera.org.uk

[48] Hopwood PE, Head ML, Jordan EJ, Carter MJ, Davey E, Moore AJ, et al. Selection on an antagonistic behavioral trait can drive rapid genital coevolution in the burying beetle, *Nicrophorus vespilloides*. Evolution. 2016;70:1180–8. 27144373 10.1111/evo.12938 PMC5089618

[49] Jarrett BJM, Schrader M, Rebar D, Houslay TM, Kilner RM. Cooperative interactions within the family enhance the capacity for evolutionary change in body size. Nature Ecol Evol. 2017;1:0178. 28685165 10.1038/s41559-017-0178 PMC5495167

[50] IBM Corp. released IBM. SPSS Statistics for Windows, Version 28.0. IBM Corp, Armonk. 2020.

[51] Trumbo ST. Interspecific competition, brood parasitism, and the evolution of biparental cooperation in burying beetles. Oikos. 1994;69:241–9.

[52] Schrempf SD, Burke KW, Wettlaufer JD, Martin PR. Behavioral dominance interactions between *Nicrophorus orbicollis* and *N. tomentosus* burying beetles (Coleoptera: Silphidae). PeerJ. 2021;9:e10797. 33665013 10.7717/peerj.10797 PMC7912668

[53] Poulin R. Explaining variability in parasite aggregation levels among host samples. Parasitology. 2013;140:541–6. 23343821 10.1017/S0031182012002053

[54] Johnson PTJ, Hoverman JT. Heterogeneous hosts: how variation in host size, behaviour and immunity affects parasite aggregation. J Anim Ecol. 2014;83:1103–12. 24548254 10.1111/1365-2656.12215

[55] Cirino BS, da Costa Neto SF, dos Santos Caro T, Maldonado Jr, Gentile R. Influence of latitude, host body size and host body weight on helminth species richness and abundance in two Neotropical marsupials. Int J Parasitol Parasit Wildl. 2025;27:101077. 10.1016/j.ijppaw.2025.101077 PMC12152593 40503084

[56] Benowitz KM, McKinney EC, Moore AJ. Difference in parenting in two species of burying beetle, *Nicrophorus orbicollis* and *Nicrophorus vespilloides*. J Ethol. 2016;34:315–9. 27917015 10.1007/s10164-016-0477-5 PMC5130308

[57] Ratz T, Smiseth PT. Flexible parents: joint effects of handicapping and brood size manipulation on female parental care in *Nicrophorus vespilloides*. J Evol Biol. 2018;31:646–56. 29468774 10.1111/jeb.13254

[58] Meierhofer I, Schwarz HH, Müller JK. Seasonal variation in parental care, offspring development, and reproductive success in the burying beetle, *Nicrophorus vespillo*. Ecol Entomol. 1999;24:73–9.

[59] Athias-Binche F. La phorésie chez les acariens. Aspects adaptatifs et évolutifs.In: Perpignan, France: Editions du Castillet; 1994;1–180.

[60] Bajerlein D, Witaliński W. Localization and density of phoretic deutonymphs of the mite *Uropoda orbicularis* (Parasitiformes: Mesostigmata) on *Aphodius* beetles (Aphodiidae) affect pedicel length. Naturwissenschaften. 2014;101:265–72. 24504532 10.1007/s00114-014-1150-x PMC3969809

[61] Pfammatter JA, Malas KM, Raffa KF. Behaviours of phoretic mites (Acari) associated with *Ips pini* and *Ips grandicollis* (Coleoptera: Curculionidae) during host-tree colonization. Agric For Entomol. 2016;18:108–18.

