# Supplementary material for: Males have a greater mite burden than females, and size does not matter: species- and sex-specific infestation patterns of mites (Uropodina) on burying beetles (Nicrophorus spp.)
Source: Front Zool. 2026 Feb 19;23:9. doi: 10.1186/s12983-026-00601-w (PMC13020040; doi:10.1186/s12983-026-00601-w)
Supplement: Supplementary file 1 — Additional file1 (DOCX 24 KB) [file 12983_2026_601_MOESM1_ESM.docx]

**Supplementary Materials**

**Males have a greater mite burden than females, and size does not matter: species- and sex-specific infestation patterns of mites (Uropodina) on burying beetles (*Nicrophorus* spp.)**

Bajerlein Daria, Zduniak Piotr, Wyszyńska Aleksandra, Baraniak Edward, Przewoźny Marek, Grzegorczyk Tomasz, Urbański Arkadiusz

**Supplementary Table 1.** Numbers of collected burying beetle species (*Nicrophorus* spp.) within particular study seasons, including their sex (M – males, F – females)

| **Beetle species** **Study season** | **2018** | | **2019** | |
| --- | --- | --- | --- | --- |
|  | May 30 – July 12 | July 13 – September 18 | May 18 – July 2 | July 3 – September 5 |
| *Nicrophorus humator* (Gleditsch, 1767) | 16 (10 F/6 M) | 275 (133 F/142 M) | 8 (4 F/4 M) | 11 (7 F/4 M) |
| *Nicrophorus vespillo* (Linnaeus, 1758) | 35 (15 F/20 M) | 290 (152 F/138 M) | 27 (9 F/18 M) | 18 (12 F/6 M) |
| *Nicrophorus interruptus* Stephens, 1830 | 42 (20 F/22 M) | 315 (138 F/177 M) | 64 (32 F/32 M) | 23 (12 F/11 M) |

**Supplementary Table 2.** Summary of the Factorial ANOVA showing results of the effect of the species, sex, and the interaction effect of species*sex on the body size in burying beetles (*Nicrophorus humator*, *N. vespillo*, and *N. interruptus*).

| **Factor** | **F** | **df** | **p** |
| --- | --- | --- | --- |
| INTERCEPT | 81676.071 | 1, 867 | < 0.001 |
| SPECIES | 262.10 | 2, 867 | < 0.001 |
| SEX | 1.01 | 1, 867 | 0.315 |
| SPECIES*SEX | 5.48 | 2, 867 | 0.004 |
